# A Qualitative Investigation of the Positive and Negative Impacts of the COVID-19 Pandemic on Post-Secondary Students’ Mental Health and Well-Being

**DOI:** 10.1177/21676968221121590

**Published:** 2022-10

**Authors:** Lexi Ewing, Chloe A. Hamza, Kaylea Walsh, Abby L. Goldstein, Nancy L. Heath

**Affiliations:** 1 Applied Psychology and Human Development, Ontario Institute for Studies in Education, 113749 University of Toronto, Toronto, ON, Canada; 2 Educational and Counselling Psychology, 153551 McGill University, Montreal, QC, Canada

**Keywords:** COVID-19, mental health, coping, emerging adulthood, post-secondary students

## Abstract

Evidence suggests that post-secondary students without pre-existing mental health concerns may have experienced worsening mental health during the COVID-19 pandemic, relative to students with pre-existing mental health concerns. To clarify the psychological impacts of the pandemic, and elucidate why differences may exist among students, 20 interviews were conducted with emerging adults enrolled in university. Using directed content analysis, eight themes were identified: three more common among students with pre-existing mental health concerns, three more common among students without pre-existing mental health concerns, and two shared. Although all students experienced novel stressors during the pandemic, students without pre-existing mental health concerns reported greater increases in social and academic isolation, relative to students with pre-existing mental health concerns. Students with pre-existing mental health concerns also leveraged existing coping repertoires, which further supported their ability to manage pandemic-related challenges. Findings highlight how postsecondary institutions can bolster student well-being.

In many developed countries, a large percentage of emerging adults (ages 18–25) enroll in post-secondary school (Arnett et al., 2014; Bureau of Labor Statistics, U.S. Department of Labor, 2020; Statistics Canada, 2019b). Typically, post-secondary education involves working toward a certificate, diploma, or degree, for which a secondary school diploma (or equivalent) is a required prerequisite (Statistics Canada, 2019a). As a result of the COVID-19 pandemic, post-secondary students worldwide have experienced a wide array of new and unprecedented challenges. These challenges have included stressors faced by the general population, such as increased social isolation stemming from social distancing guidelines, lockdowns, and stay-at-home orders (Hotez et al., 2021; Kujawa et al., 2020). In addition, students have experienced stressors unique to the post-secondary context. These have included navigating the transition to online learning, as well as loss of practicum and employment opportunities (Farris et al., 2021; Son et al., 2020; Vuletić et al., 2021). The pandemic also has led to disruptions in normative developmental tasks for emerging adults, such as identity exploration and developing new social and romantic relationships (Halliburton et al., 2021; Son et al., 2020; Vuletić et al., 2021). In the present study, we sought to understand how the pandemic, and resulting changes for post-secondary students, impacted student psychological health and well-being.

## The Psychological Impacts of the COVID-19 Pandemic

At the onset of the COVID-19 pandemic, many authors initially cautioned that students may be particularly vulnerable to the psychological impacts of the pandemic (Cao et al., 2020; Lederer et al., 2021; Pierce et al., 2020; Son et al., 2020). However, mounting evidence suggests that the mental health impacts of the COVID-19 pandemic on post-secondary students may not be as severe as originally expected. Some cross-sectional studies have demonstrated higher levels of psychological distress among post-secondary students compared to the general population during the pandemic (Kibbey et al., 2021; Odriozola-González et al., 2020; Xiong et al., 2020), but students were already at higher risk for mental health concerns prior to the pandemic (American College Health Association, 2019; Auerbach et al., 2016; Oswalt et al., 2020). Further, longitudinal research that has examined changes in the prevalence of mental health concerns prior to and during the pandemic in Europe, Asia, North America, and the Oceania, suggests that the psychological impacts of the COVID-19 pandemic may be quite modest (for a review, see Prati & Mancini, 2021). For example, in longitudinal studies of emerging adults living in Canada and The Netherlands, average levels of depression and anxiety symptoms were fairly stable, at least in the early months of the pandemic. A key emergent finding in the literature that should be considered though, is there seems to be significant variability in risk, with some individuals more or less at-risk of elevations in mental health symptoms than others (van den Berg et al., 2021; van Zyl et al., 2021; Watkins-Martin et al., 2021). This work has led authors to conclude that impacts of the COVID-19 pandemic may not be uniformly detrimental (Prati & Mancini, 2021; Watkins-Martin et al., 2021).

To better understand variability in psychological responses to the pandemic, researchers have turned their attention to studying factors associated with psychological vulnerability during the COVID-19 pandemic. In the general population, loss of a loved one due to COVID-19 and having a recent positive case in immediate social networks have been associated with heightened risk for distress (Kibbey et al., 2021; Li et al., 2021; López-Castro et al., 2021). The COVID-19 pandemic also has amplified existing health inequalities. Living with a chronic illness, or being at high risk for severe disease, have been linked to heightened psychological vulnerability (Browning et al., 2021; Kibbey et al., 2021; Xiong et al., 2020). Ethnically or racially minoritized groups, as well as those experiencing socioeconomic disadvantage, also have been found to be at higher risk for increasing distress during the pandemic (Browning et al., 2021; Iob et al., 2020; Mathias et al., 2020; Ray et al., 2021). This heightened risk among marginalized groups may stem from increased risk of COVID-19 exposure, as a result of residential crowding, lack of access to outdoor spaces, greater use of public transit, as well as more essential service work (Patel et al., 2020; Smith & Judd, 2020). Relatedly, greater job loss, food, and housing insecurity during the pandemic may serve as significant stressors (Browning et al., 2021). In addition, lack of access to physical and mental health care, and poorer quality care stemming from discrimination may also contribute to the higher rates of stress, morbidity, and mortality among marginalized groups (Golestaneh et al., 2020).

## Prior Mental Health and Psychological Vulnerability During the COVID-19 Pandemic

An individual’s mental health prior to the pandemic may also be an important factor for understanding vulnerability for distress during the pandemic. At the onset of the pandemic, many authors cautioned that individuals with pre-existing mental health concerns may be most at-risk (Druss, 2020; Yao et al., 2020). Contrary to expectations, in post-secondary and emerging adult samples, some studies suggest that individuals with pre-existing mental health concerns have shown stability in mental health over time. In contrast, individuals without pre-existing mental health concerns have shown declining mental health (Hamza et al., 2020; Meda et al., 2021; Watkins-Martin et al., 2021). For example, Hamza et al. (2020) found that during the COVID-19 pandemic, Canadian post-secondary students without pre-existing mental health concerns showed worsening stress, depressive, and anxious symptoms compared to 1 year prior to the pandemic. There was stability, or even improving mental health, for individuals with pre-existing mental health concerns. Similar patterns have also been found among individuals in the general population (Czysz et al., 2021; Fancourt et al., 2021; Pan et al., 2021; Pinkham et al., 2020).

It is less clear why students without pre-existing mental health concerns may be more vulnerable to the psychological impacts of the COVID-19 pandemic than students with pre-existing mental health concerns. In a sample of American adults with a history of depressive symptoms, Czysz et al. (2021) suggested that the stability in mental health indicators may be due to a ceiling effect, such that individuals with pre-existing concerns have little room for worsening mental health symptomology. Alternatively, it has been suggested that individuals with pre-existing mental health concerns may be more equipped for the life changes associated with the COVID-19 pandemic. This may be due to previous experience managing difficult emotions or due to having existing support structures in place (i.e., already accessing professional mental health treatment) (Czysz et al., 2021; Hamm et al., 2020; Murphy et al., 2021). Further, social distancing guidelines may have been less impactful for students with pre-existing mental health concerns, given that they may have already been experiencing greater social isolation prior to the pandemic than individuals without pre-existing mental health concerns (Hamza et al., 2020).

## The Present Study

The aim of the present study was to clarify the psychological impacts of the COVID-19 pandemic on post-secondary students with and without pre-existing mental health concerns. Based on findings from a larger quantitative study, we anticipated that students without pre-existing mental health concerns would report declining mental health in the context of the pandemic. In contrast, we expected that students with pre-existing mental health concerns would show more stability in mental health. Using a directed qualitative approach, we sought to illuminate from participants’ perspectives if, and why, these groups may have had different experiences during the pandemic. Specifically, students with and without pre-existing mental health concerns from one large urban university were interviewed about their experiences during the COVID-19 pandemic. At the time of the interviews, all participants were living in Toronto, Ontario under a government mandated state of emergency, and a series of public health measures were in place (e.g., stay-at-home orders) (Government of Ontario, 2021). Given that it has been suggested that the psychological impacts of the pandemic will likely continue to persist after the pandemic has peaked (Fiorillo & Gorwood, 2020; Galea et al., 2020; Gunnell et al., 2020), it is important to understand the enduring impacts of the COVID-19 pandemic on students. Moreover, understanding which students are most vulnerable, and why, can support ongoing targeted intervention for at-risk students on post-secondary campuses.

## Methods

### Study Design

Guided by a previous quantitative study exploring students’ experiences prior to and during the COVID-19 pandemic (Hamza et al., 2020), the current study employed a deductive approach to explore the nuanced experiences of students with and without pre-existing mental health concerns during the COVID-19 pandemic. This study utilized directed content analysis (DCA), which is a qualitative method supporting the identification of themes and patterns within the data that are informed by previous research findings. This approach is useful when research on a phenomenon could benefit from further in-depth description (Hsieh & Shannon, 2005).

### Research Team Positionality

The authors note several aspects of positionality in recognition of how researcher identities can influence qualitative data collection, interpretation, and analyses. All authors identify as White cis-gendered women. LE and KW are graduate students studying post-secondary student mental health. CH, AG, and NH are full-time faculty members with extensive clinical and research expertise in mental health during emerging adulthood. All five authors are involved in mental health advocacy in post-secondary contexts, and work closely with individuals with lived experience. LE, CH, AG, and NH were involved in a larger quantitative study on the psychological impacts of COVID-19 for students with and without pre-existing mental health concerns, for which this study served as a follow-up. All of the authors have advanced training in ethics, psychological research and practice, and research methods. Prior to conducting the interviews, LE completed advanced course work in qualitative methods, conducted practice interviews with CH, and completed suicide risk assessment training. Throughout the qualitative research process all authors reflected on their positionality and were mindful of how their identities, their own mental health, and their experiences with post-secondary educational systems, may influence their understanding and interpretation of the results.

### Sample

In total, 20 post-secondary students participated in the present study, of which 10 were identified as having a pre-existing mental health concern and 10 were identified as having no pre-existing mental health concerns (see Table 1). Participants for the present qualitative study were drawn from a larger longitudinal quantitative research study focused on stress and coping in post-secondary school. Both studies were conducted with students enrolled at the University of Toronto, a large academically rigorous Canadian university. To be eligible for the larger quantitative study, participants had to be first-year students, fluent in English, and reside in the city in which the university was situated (Toronto, Ontario). The same eligibility criteria extended to the present qualitative study, though participants could be enrolled in any year of their undergraduate studies at the University of Toronto (i.e., inclusion was not restricted to first-year students).

**Table 1.** Participant Demographics by Pre-Existing Mental Health Concern Status*.*

Participant Number	Age	Gender	Depressive Symptoms(Cut-Off = 22)	Anxiety Symptoms (Cut-Off = 10)	Borderline Personality Disorder Characteristics (Cut-Off = 7)	Alcohol Dependence Symptoms(Cut-Off = 8)
Pre-existing mental health concern						
Participant #1	19	Female	52	21	9	4
Participant #2	19	Female	49	21	8	0
Participant #3	20	Female	28	14	4	0
Participant #4	19	Female	41	21	7	2
Participant #5	20	Female	26	12	3	0
Participant #6	20	Female	45	11	7	3
Participant #7	19	Female	37	12	7	1
Participant #8	19	Female	25	6	3	18
Participant #9	19	Male	36	0	0	0
Participant #10	19	Male	40	17	8	1
No pre-existing mental health concern						
Participant #11	18	Female	13	4	5	0
Participant #12	20	Female	19	2	3	3
Participant #13	19	Female	21	4	3	1
Participant #14	20	Female	11	3	4	0
Participant #15	19	Female	20	9	4	5
Participant #16	21	Male	14	2	1	0
Participant #17	20	Male	1	0	0	5
Participant #18	19	Female	14	2	5	3
Participant #19	19	Female	14	4	4	0
Participant #20	20	Male	20	3	2	6

*Note.* Depressive symptoms were scored with the Centre for Epidemiologic Studies Depression Scale – Revised (CESD-R), anxiety symptoms were scored with the Generalized Anxiety Disorder-7 (GAD-7), borderline personality disorder characteristics were scored with the McLean Screening Instrument for Borderline Personality Disorder (MSI-BPD), and alcohol dependence symptoms were scored with the Alcohol Use Disorders Identification Test (AUDIT).

At the time of study enrollment, participants were 18–21 years old (*M*age = 19.40, SD = .68) and 75% (*n* = 15) identified as female. Thirty-five percent (*n* = 7) of the participants identified as East Asian, 30% (*n* = 6) identified as South Asian, 20% (*n* = 4) identified as White, 10% (*n* = 2) identified as Filipino, and 5% (*n* = 1) identified as Black. Sixty percent (*n* = 12) of participants were living with their parents, 25% (*n* = 5) were living with roommates/friends, and the remaining 15% (*n* = 3) were living either alone or with a partner. Overall, 65% (*n* = 13) of participants came from households where both parents received a university degree or higher. Participant demographics are representative of the population in Toronto, Canada, where all participants were living during the study. As Canada’s largest city, Toronto is a demographically diverse population. Over half of the population identifies as a visible minority and 52% are immigrants. Most of the population are enrolled in, or have completed, post-secondary education and 49.9% are employed full-time. Household income is largely varied, with 20.2% of the population classified as low-income and 10.5% of the population earning over $100,000CAD each year (Statistics Canada, 2017).

As part of the larger longitudinal quantitative study, participants completed several online survey measures about their mental health in May 2019 prior to the COVID-19 pandemic, and in May 2020 during the COVID-19 pandemic. Using participants’ responses from the May 2019 survey, previously established cut-off scores were utilized to identify participants with and without pre-existing mental health concerns. Participants had to meet the criteria for at least one of the following, including a cut off score of 22 on the Centre for Epidemiologic Studies Depression Scale - Revised (CESD-R; Eaton et al., 2004; Van Dam & Earleywine, 2011), a cut-off score of 10 for the Generalized Anxiety Disorder questionnaire (GAD-7; Spitzer et al., 2006; Sriken et al., 2022), a cut-off score of 7 on the McLean Screening Instrument for Borderline Personality Disorder (MSI-BPD; Gardner & Qualter, 2009; Zanarini et al., 2003), and/or a cut-off score of 8 on the Alcohol Use Disorders Identification Test (AUDIT; Hagman, 2016; Saunders et al., 1993). Of the ten students who met our criteria for pre-existing mental health concerns, all of the students reported clinically significant depressive symptoms, 80% (*n* = 8) reported clinically significant anxiety symptoms, 60% (*n* = 6) reported clinically significant BPD symptoms, and 10% (*n* = 1) reported clinically significant alcohol use disorder symptoms. Ninety percent (*n* = 9) of the subsample had co-occurring mental health concerns, such that they met the clinical cut-off criteria of two or more indicators.

In Winter 2021, the primary author reached out to participants in each of these two groups who consented to be contacted about follow-up study opportunities. In total, 44 students from the pre-existing mental health concern group were emailed and/or phoned and invited to participate in an online interview, and 10 agreed to participate. Another 29 students for the no pre-existing mental health concern group were invited, and 10 agreed to participate. Although some participants indicated they were not interested in the follow-up study (*n* = 8), low response rates also were a result of being unable to reach some students with the contact information provided the year prior (*n* = 40), as well as students having relocated outside of the city in which data was collected (*n* = 5). Students could not participate if they did not reside in the city in which data was collected due to ethical concerns about being able to connect students with local resources if needed. These recruitment challenges were likely attributable in part to frequent changes in living arrangements and housing for students stemming from the COVID-19 pandemic (Sahu, 2020). There were no significant differences on quantitative study variables assessed in the May 2019 survey between students who agreed to participate and those who were not interested or were unable to participate. Participant recruitment occurred over a 2-month period, as interviews were conducted, and additional students were recruited until data saturation was reached for both groups.

### Data Collection

This study was approved by the University of Toronto research ethics board (Protocol # 40063). Prior to scheduling an interview, each student who responded to the study invitation was provided an information sheet about the study. Students who indicated they wanted to participate were then scheduled to partake in an interview online via Microsoft (MS) Teams. To maintain participant confidentiality during the MS Teams recording, all students were assigned a unique ID number and were provided with a one-time password protected email account to complete the interview. Before the interview, participants were required to provide written consent, and parameters of confidentiality were reviewed again at the beginning of the interview. All interviews were conducted by the primary author (LE).

A semi-structured interview guide was established by all five authors (LE, CH, KW, AG, NH) to provide standardization of questions. This process involved meeting to discuss the questions, drafting and editing the questions, and coming to consensus on the interview questions and prompts. Both groups (i.e., students with pre-existing mental health concerns and students without pre-existing mental health concerns) were asked the same set of questions during the interview. The interview questions focused on students’ experiences during the COVID-19 pandemic, the specific stressful events that occurred, and how they coped with these events. Although some of the questions were more open-ended (e.g., “So to start, can you tell me a bit about your experiences so far during the COVID-19 pandemic, and how things have been going for you generally?”), more directed questions were also asked to examine central study aims (e.g., “Would you say there has been a change in the stressors you experienced prior to, compared to now during, COVID-19?” “Can you describe how you specifically coped with these stressful experiences?”). Throughout the interview additional follow-up and probing questions were used to encourage students to expand upon their initial responses (e.g., “Could you tell me more about that experience?”). For the purposes of consistency, the interview guide did not evolve over the course of the interviews, and the complete guide can be requested from the corresponding author. The interviews ranged from 20 – 60 minutes in length, depending on students’ responses, and students received a $10 gift card for participating. Interviews were recorded via MS Teams and immediately transcribed via NVivo Transcription Services.

Although research has consistently found that asking young adults to report on their mental health does not have any associated iatrogenic effects or lead to increased psychological distress (Dazzi et al., 2014), several precautions were taken. At the end of the interview, all students were provided with a comprehensive list of local mental health agencies to contact if they experienced any distress. Students were also told that they could access this list of supports at any point during the interview, that they could choose to not answer questions they were uncomfortable with, and that they could withdraw from the interview at any time. Finally, a crisis response protocol developed by CH and AG was in place, in the event any suicidal ideation or behaviors were disclosed during the interview.

### Data Analysis

The data analysis was guided by the recommended DCA approach described by Assarroudi and colleagues (2018), and consisted of three phases including preparation, organization, and reporting of findings (Elo & Kyngas, 2008).

#### Phase one: preparation

The initial preparation phase involved familiarization with the findings from a previous quantitative investigation of student experiences during the COVID-19 pandemic (Hamza et al., 2020), selection of the stratified random sampling strategy and interview guide development, conducting and transcribing the interviews (the unit of analysis) verbatim, and immersion in recordings and interview transcripts. Given that interviews were conducted online, no participant visual information was recorded to maintain participant confidentiality. As a result, analyses focused on manifest interview content only (i.e., students’ words, rather than nonverbal language).

#### Phase two: organization

The organization phase included deductively identifying initial categories derived from results of the previous study (Hamza et al., 2020). New patterns identified from the interview data were then inductively categorized and defined based on principles of thematic analysis (Clarke & Braun, 2017). Two researchers (LE and KW) independently reviewed and coded all 20 interviews in NVivo 12 and met weekly to review codes and memo notes. Individual quotations coded into each category were examined and summaries of the patterns represented within each category were developed to create themes. Interviews were conducted until data saturation was reached (Francis et al., 2010). Data saturation was considered to be reached when no new concepts were obtained from the interviews either deductively or inductively (Assarroudi et al., 2018; Cleary et al., 2014). Before proceeding to the reporting phase, the themes were reviewed by LE, CH, and KW to determine the final set of findings. A thorough audit trail was maintained through the organization phase.

#### Phase three: reporting

The final reporting phase consisted of information presented in the present manuscript, including a detailed description of the methodology and study findings. Additionally, given that directed content analysis is informed by previous research findings it is crucial to employ certain steps to reduce authors’ pre-existing biases. To ensure trustworthiness, the authors utilized the 16-step method developed by Assarroudi and colleagues (2018), as well as the trustworthiness checklist developed by Elo and colleagues (2014). Both tools map on to the three stages of directed content analysis (Elo & Kyngas, 2008) and support the five criteria of trustworthiness in qualitative research: credibility, confirmability, authenticity, dependability, and transferability (Lincoln & Guba, 1985). Examples of trustworthiness techniques employed included data saturation (credibility), audit trails (confirmability), quotations from multiple participants (authenticity), and a detailed description of study methodology and sampling strategy (dependability and transferability) (Kyngäs et al., 2019). Additionally, an intercoder reliability test using Cohen’s kappa statistic was calculated in NVivo 12 to assess the level of agreement between the two coders, and good agreement was found for all themes (Mikkonen & Kyngäs, 2019).

## Results

**Figure 1.**
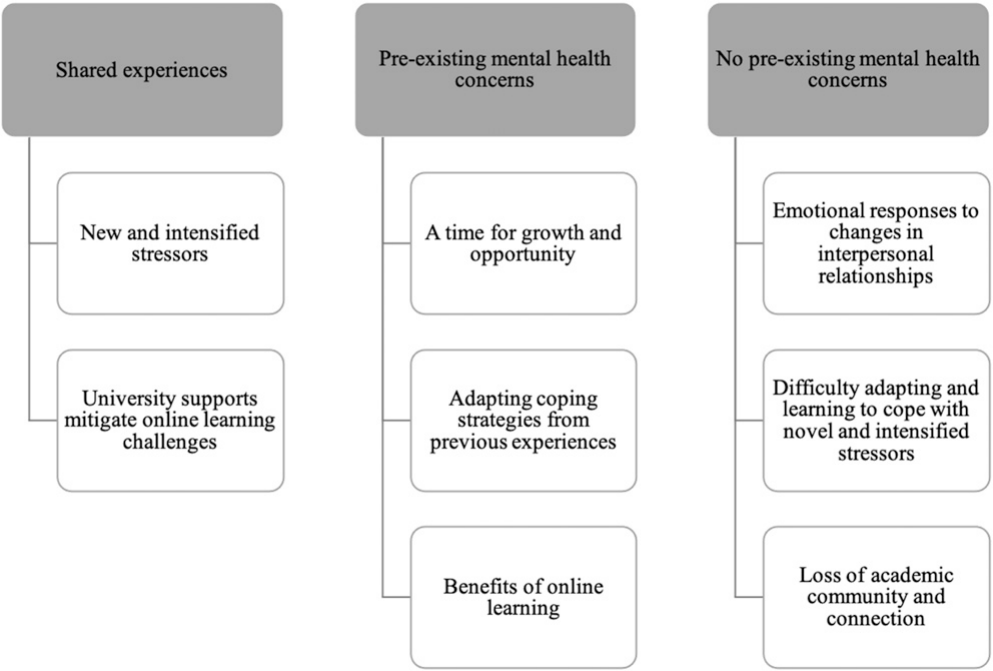
Themes by pre-existing mental health concern status.

Students discussed a range of experiences during the COVID-19 pandemic, which provided insight into the differential impact of the pandemic on post-secondary students with and without pre-existing mental health concerns. Eight themes were identified; two themes were shared by both students with and students without pre-existing mental health concerns; three were more central to students with pre-existing mental health concerns, and three were more central to students without pre-existing mental health concerns (see Figure 1). In theme descriptions, the use of “many” refers to ideas mentioned by 50% or over of the relevant sample and the use of “some” refers to ideas mentioned by under 50% of the relevant sample.

### Shared Experiences Among Students With and Without Pre-Existing Mental Health Concerns

#### New and intensified stressors (n = 20)

All students spoke about new stressors due to the COVID-19 pandemic that they felt were not concerns prior to the onset of the pandemic. The most common novel stressor indicated by students was substantial worry about their own health and the health of their loved ones due to the unpredictability and novelty of the COVID-19 virus. The distress associated with the COVID-19 virus also brought about new stressors related to COVID-19 restrictions (i.e., stay-at-home orders) and social disagreements with close friends. As indicated by one participant, “It’s been hard because I feel like I have to … distance myself from the people who are not taking it seriously … in order to be safe and protect the people that I do see.” [Participant 18, No pre-existing mental health concern, age 19]. Further, many participants also spoke to experiencing increased distress in response to existing stressors as their coping abilities were more taxed due to the pandemic, as articulated by one participant, “I feel like the stressors are the same, but the amount of weight that they stress me out has changed.” [Participant 11, No pre-existing mental health concern, age 18].

#### University supports mitigate online learning challenges (n = 19)

The majority of students across both groups spoke to the benefits of university supports during this unprecedented time. Participants identified broad supports from the university, ranging from increased institutional-provided mental health support to policy changes (i.e., credit/no credit courses). As indicated by one participant: “[in the shift to online school, the university] really supported me, so I thank them for that” [Participant 20, No pre-existing mental health concern, age 20]. Further, students often spoke to a change in professor support during the COVID-19 pandemic compared to prior to the COVID-19 pandemic. Students indicated that professors were more understanding of how external factors influence academic success and readiness, and also provided more opportunities for students to re-take tests and re-submit assignments:

“I feel like the, at least the professors I have, not all of them, but some of them are very, very open to kind of discussing, you know, the things that you can rearrange or if you need help with something or if you want to move a deadline. In my experience, they’ve been very, very welcoming to those requests, not always, but generally, which I, which I find is different than before. I feel like before those of kind of guidelines and standards were a little more strict.” [Participant 10, Pre-existing mental health concern, age 19].

### Experiences More Common Among Students With Pre-Existing Mental Health Concerns

#### A time for growth and opportunity (n = 9 students with pre-existing mental health concerns)

Many students with pre-existing mental health concerns indicated that the increased time available, including more time alone, helped with their self-development and growth. They reported more opportunities to focus on further developing positive relationships with themselves and with others. Often, students highlighted that because other opportunities were not available, they were forced to find other ways to spend time, as indicated by this participant:

“I think just having time and just like being forced to not overextend myself because everything is closed and so I didn’t have to, I didn’t have to think about going to work or I didn’t have to throw myself at a bunch of extracurriculars … So just not having the option to keep myself busy with a whole bunch of other things definitely gave me the time to just focus on making myself feel better and working on my relationships and stuff.” [Participant 7, Pre-existing mental health concern, age 19].

Further, for students with pre-existing mental health concerns, the increase in time spent alone was a catalyst for developing confidence in their own skills to manage difficult emotions and “gave [them] time to focus on that mental health distress and address it” [Participant 1, Pre-existing mental health concern, age 19]. Some of these participants indicated that before the COVID-19 pandemic, they relied heavily on interpersonal relationships to navigate challenging situations. However, with these relationships not readily available, primarily due to public health restrictions, they learned to develop these strategies within themselves, as demonstrated by this participant:

“Before COVID-19, I just kept on talking to my friends … I expected them to help me out with my emotions and I’d be like, well, what should I do? I feel bad about this. I am trying, but nothing’s helping. And then they would be like just keep on trying, just keep on trying, it’s going to get better. Even though I knew it’s me that would have to change. It’s not their words that change me. So that’s one thing that really changed me. Like, after, during COVID, I realized like, oh, it’s me. I have to change. It’s not the friend, not talking to friends, that change me.” [Participant 5, Pre-existing mental health concern, age 20].

It is important to highlight that although these students still found the COVID-19 pandemic to be difficult, they seemed to find it less challenging than participants without pre-existing mental health concerns, as Participant 10 indicates: “I do still see [the COVID-19 pandemic] as an obstacle. But I think regardless of everything, kind of for me, because I have previously struggled, struggled with like mental health issues, I think for me it’s not necessarily the worst thing that has happened.” [Pre-existing mental health concern, age 19].

#### Adapting coping strategies from previous experiences (n = 8 students with pre-existing mental health concerns)

When asked about how they dealt with stressors related to the COVID-19 pandemic, many students with pre-existing mental health concerns felt like they were able to navigate stressors more successfully, whereas students without pre-existing mental health concerns did not often discuss their ability to manage COVID-19 stressors well (*n* = 3 students without pre-existing mental health concerns endorsed this theme). Students with pre-existing mental health concerns primarily attributed their management of COVID-19 stressors to previous experiences that they had managing difficult emotions, as indicated by this participant:

“I knew going into the pandemic that, you know like, I don’t know how long this is going to last and I kind of need to be prepared for anything like knowing what, how I react previously to situations that I don’t really have control over. So I kind of like knew what to expect in terms of my personal reaction and that kind of helped me going in on immediately, kind of looking for ways to cope with it.” [Participant 1, Pre-existing mental health concern, age 19].

These students often mentioned that because they had previously experienced overwhelming distress, they were well equipped to deal with the complex and sudden emotions arising from the COVID-19 pandemic. For example, students indicated that they had the tools to navigate difficult emotions and knew the importance of addressing overwhelming emotions urgently. As one participant articulated, previous experiences with mental health concerns may provide a useful knowledge base for managing novel and distressing major life events:

“I was going through some stuff before like before lockdown, so … I would have to say, like people, maybe like we were better equipped to deal with changes like this because we dealt with, like, difficulties before compared to other students who haven’t dealt with, like depression or like bad anxiety” [Participant 8, Pre-existing mental health concern, age 19].

Interestingly, a few students with no pre-existing mental health concerns (*n* = 3) also suggested that students with previous experience with mental health difficulties may be better prepared to deal with emerging and novel COVID-19 stressors due to previous experiences working through difficult emotions and challenging life events. As described by Participant 18, a student with no pre-existing mental health concerns:

“Maybe people who are experiencing mental health difficulties, you know, they’ve kind of, they might have their toolkit for dealing with issues or they might be more resilient and things like that, whereas maybe people who haven’t had to think about that before are kind of just thrown into this traumatizing situation.” [Participant 18, No pre-existing mental health concern, age 19].

#### Benefits of online learning (n = 6 students with pre-existing mental health concerns)

Many students with pre-existing mental health concerns spoke to finding benefit in the transition to online learning environments. This was primarily attributed to time gained from not having to commute or travel across campus for classes during the school day. Some students explained that online learning enhanced their comfort participating in class, mainly due to having more participation options (i.e., text-responses in a chat box, anonymous polling) or being able to attend class from the comfort of their own home as Participant 7 explains:

“I had a lot of anxieties around being around people before the pandemic and just being super self-conscious. But because everything’s online, I always have the option to mute myself or turn off my video whenever I feel uncomfortable, or I just want a minute to myself, or I want to go get a snack. And it just gave me this sense of comfort I suppose. And I’m sure I’m not the only one that has felt like that. So, I suppose being online has certainly created this level of comfort.” [Pre-existing mental health concern, age 19].

This time spent learning online may also help facilitate enhanced participation when moving back to in-person, as articulated by one student:

“It’s definitely made me feel like more confident like maybe, hopefully, when everything is in person again, I can buy that same confidence and actually say what I’m thinking during my labs and tutorials and stuff. But yeah, it’s definitely been better for me.” [Participant 6, Pre-existing mental health concern, age 20].

### Experiences More Common Among Students Without Pre-Existing Mental Health Concerns

#### Emotional response to changes in interpersonal relationships (n = 9 students without pre-existing mental health concerns)

Students with no pre-existing mental health concerns spent considerable time during the interviews talking about the changes to interpersonal relationships during the COVID-19 pandemic, whereas students with pre-existing mental health concerns highlighted interpersonal changes less frequently (*n* = 4 students with pre-existing mental health concerns endorsed this theme). Students with no pre-existing mental health concerns expressed feeling sadness due to loss of in-person socialization and highlighted that virtual socialization is “not the same as talking to [friends] physically and being there with that person” [Participant 19, No pre-existing mental health concern, age 19]. Some students with no pre-existing mental health concerns also indicated that COVID-19 restrictions negatively impacted their ability to fully live out their post-secondary years through social gatherings or exploring a new city.

“I kind of feel like … my college experience was like ruined per se … I look back at my parents and like kids before me and they got a great experience and like, there’s nothing I can really do about it.” [Participant 17, No pre-existing mental health concern, age 20].

Throughout the interviews, students with no pre-existing mental health concerns indicated that in-person connections were important for the strength and fulfillment of their relationships, and expectations of their current life stage. As mentioned in other themes, the isolation stemming from the COVID-19 pandemic and associated restrictions was particularly concerning for those with no pre-existing mental health concerns.

“I’m a fairly extroverted person, and so you could say I thrive in, when I’m surrounded by people, or like going out with my friends or just going, leaving the house. But now that I’m sort of stuck at home forcefully, it’s been kind of hard on me.” [Participant 14, No pre-existing mental health concern, age 20].

#### Difficulty adapting and learning to cope with novel and intensified stressors (n = 8 students without pre-existing mental health concerns)

Many students with no pre-existing mental health concerns felt that they had a difficult time managing negative emotions and mitigating stressful events related to the COVID-19 pandemic. This was primarily driven by students feeling that they did not experience many major stressors prior to the COVID-19 pandemic, nor were they aware of the influence that these stressors may have had on their emotions: “I think prior to COVID-19, I didn’t have as much stress or if I did, I didn’t like realize it as much” [Participant 14, No pre-existing mental health concern, age 20]. When asked to explain how students previously dealt with stress when it did arise, many students with no pre-existing mental health concerns spoke to communal stress management, whereby stress was reduced when they felt others were experiencing similar emotions, as indicated by one participant:

“I feel like before um, I didn’t really need, like, a way to deal with stuff because … you’re like around a lot of your peers all the time that are going through the same stressors. And then just like little things, like standing outside together before an exam, and you’re all talking about how nervous you are, like, makes you feel less nervous because, you know, everyone’s going through the same things.” [Participant 13, No pre-existing mental health concern, age 19].

Further, students indicated that before the COVID-19 pandemic they would often avoid thinking about and/or managing difficult emotions, which resulted in little prior experience actively managing stress: “I’ve always kind of dealt with stressors in the same way … like I try to not face what’s bothering me, which also doesn’t really help” [Participant 11, No pre-existing mental health concern, age 18]. With the increased stress brought on by the pandemic, and limited access to the usual channels for managing stress (i.e., communal stress management), many students with no pre-existing mental health concerns did not feel well-equipped to manage novel and intensified stressors during the COVID-19 pandemic. Importantly, these students indicated that while they had difficulty learning to cope with stressors, they were slowly learning and employing effective strategies over the course of the pandemic:

“I think that I have, I’ve developed ways of dealing with stress that I know work that I didn’t really have before COVID because, I don’t know, there were just so many distractions that that was kind of like my way of dealing with stress. But now that there are not those distractions, I’ve had to develop ways to deal with it. And now that I know those ways, I can put them into action when I do get stressed.” [Participant 17, No pre-existing mental health concern, age 20].

Interestingly, some students with no pre-existing mental health concerns indicated that they spoke with close friends who had previous experience with mental health concerns to learn how best to manage difficult feelings, and that these conversations were helpful: “I have some friends who have struggled with mental health issues before COVID. And so, it’s been kind of like following what they say.” [Participant 14, No pre-existing mental health concern, age 20].

#### Loss of academic community and connection (n = 8 students without pre-existing mental health concerns)

Students with no pre-existing mental health concerns commonly spoke about the loss of academic community and connection due to the sudden transition to online learning. Online learning resulted in the inability to socialize with peers enrolled in the same course, which was an important aspect to both students’ enjoyment of courses and to their sense of success with course content. As explained by one participant:

“But now [with courses online], if you don’t know anyone in a class like, you’re kind of screwed because there’s not really any way to meet anyone else. And sometimes when you’re doing assignments and stuff you just have no idea what you’re doing and … there’s no one to reach out to. But if it was in person … if you’re, like, really desperate, you can just ask someone sitting beside you. But it’s a lot more isolated now.” [Participant 13, No pre-existing mental health concern, age 19].

This lack of connection also made some students feel as though they were alone with their academic stress, which influenced their motivation to complete assignments and course readings. Additionally, some students felt that the online learning environment made information retention and understanding more difficult. As one participant articulates:

“There’s just something about being right next to somebody who’s trying to teach you something and like everyone being in that room, that just changes it. It just changes you. Like you just focus better. You just have more like knowledge being, like, distributed throughout the classroom. And like, everyone just kind of knows what’s going on. Everyone’s on the same page. But online, it just, I don’t, I don’t know. It just doesn’t feel right. It just doesn’t, I have to watch a lecture like two times to, like, really let it sink.” [Participant 16, No pre-existing mental health concern, age 21].

The decreased motivation and difficulty learning contributed to a build-up of work, which in turn made students feel more stressed.

## Discussion

The aim of the present study was to extend emerging findings that post-secondary students with and without pre-existing mental health concerns in Ontario, Canada may vary in their psychological responses to the pandemic (Hamza et al., 2020). Using a directed qualitative approach, eight key themes were identified to capture participants’ lived experiences during the pandemic. Several important differences emerged between students with and without pre-existing mental health concerns. Specifically, in this predominantly female sample of East Asian, South Asian and White emerging adults from middle to upper-class backgrounds at the University of Toronto, we found that students with pre-existing mental health concerns reported being able to leverage existing coping repertories more effectively. In contrast, students without pre-existing mental health concerns struggled to adapt previous strategies (e.g., drawing on social supports), and seemed to find reduced social interactions and the transition to online learning more challenging relative to students with pre-existing mental health concerns. Our findings align with emerging evidence that students without pre-existing mental health concerns may have been more at risk for worsening mental health during the pandemic than students with pre-existing mental health concerns (Hamza et al., 2020; Meda et al., 2021; Watkins-Martin et al., 2021), given heightened stress and challenges with coping among these students in the context of the pandemic.

### The Pandemic as a Time of New And Intensified Stressors For Post-Secondary Students

Generally, all students spoke to novel stressors related to the COVID-19 virus itself, including worry about the health of loved ones and the self, concern of infecting others, and unpredictability of future virus mutations. These virus-specific concerns are consistent with broader literature from the general population and provide support for the pervasive and unique stress that stems from the unprecedented nature of the pandemic (Farris et al., 2021; Kujawa et al., 2020; Vuletić et al., 2021). New stressors specific to the post-secondary context were also discussed by students. Students were concerned about delays in degree completion and perceived decreases in quality of learning; worries that they did not often consider prior to the pandemic. New social stressors also occurred given COVID-19 public health measures (i.e., stay-at-home orders), primarily related to conflicts with peers due to disagreements in following and/or supporting COVID-19 restrictions. Not only did students indicate new stressors associated with the COVID-19 pandemic, but many students also suggested that the severity of existing stressors increased (e.g., increased conflict with parents as a result of more time spent at home). These findings suggest that working to mitigate stressors stemming from the pandemic and helping students to cope with stressors (e.g., conflict resolution with peers, degree contingency planning), may be important ways institutions can support students in the context of the pandemic.

### Prior Experience Managing Distress Fostered Resilience During the Pandemic

Our findings suggest that prior experiences coping with distress proved beneficial in the context of the COVID-19 pandemic. Notably, one distinguishing feature of the experiences of students with pre-existing mental health concerns was their perceived ability to adapt to COVID-19 stressors and to manage associated emotional outcomes (i.e., increased distress). This confidence was largely attributed to having previous experience managing difficult emotions and being able to identify strategies that could be used to mitigate these stressors. These findings suggest that many of these students may have had an existing repertoire of coping strategies to draw on, and that these strategies continued to be accessible in the context of the pandemic. Hamm et al. (2020) reported a similar finding among a sample of older adults with pre-existing Major Depressive Disorder, showing that effective coping during COVID-19 was possible when individuals had knowledge of how to practice self-care when distressed, and access to mental healthcare and social supports. These results are also in line with transactional models of coping, which suggest that coping strategies are developed and refined in response to encounters with events an individual perceives as stressful (Zimmer-Gembeck & Skinner, 2016). Students with pre-existing mental health concerns may have developed effective coping strategies due to their previous experiences managing difficult emotions and could leverage these strategies when pandemic-related challenges arose.

In contrast, students without pre-existing mental health concerns seemed to have a challenging time learning to mitigate distress stemming from the pandemic. Not only did these students mention more frequent and severe stressors, but they also indicated difficulty in learning how to effectively cope with emotions arising from these stressors. The perceived difficulty managing distress was primarily related to limited access to prior coping strategies, such as communal stress management and in-person socialization. Across many interviews, students indicated that in-person, interpersonal interactions (e.g., going out for dinner with friends, standing with peers before an exam) played a large role in their ability to manage stress prior to the pandemic. Although this was not something they were acutely aware of previously, it became apparent once this coping strategy was no longer available to them. Restricted access to coping strategies that previously helped them to effectively manage distress left these students feeling unsure how to cope, specifically during the beginning of the pandemic. Students without pre-existing mental health concerns also attributed their perceived difficulty adapting coping strategies to less experience actively managing overwhelming difficult emotions in response to uncontrollable stressors. Though students without pre-existing mental health concerns had experienced challenges, these experiences were more limited relative to their peers with pre-existing mental health concerns. Together, these results suggest that an individual’s life experiences, in part informed by their pre-existing mental health status, influence their perceived ability to cope with stress during the pandemic.

### Students Experienced Changing Social and Academic Contexts Differentially

Most students acknowledged that changes in social and academic life resulting from the pandemic were impactful, but students without pre-existing mental health concerns perceived these changes as more negative than students with pre-existing mental health concerns. Though isolation stemming from the COVID-19 pandemic has been consistently identified as a significant contributor to mental health deterioration in the literature (Elmer et al., 2020; Hamza et al., 2020; Patterson et al., 2021), it may have been more impactful for students without pre-existing mental health concerns. These students indicated heavily valuing in-person connections derived from both social and academic contexts, and often discussed how integral in-person connection was to their well-being and academic success. It is possible that feelings of isolation resulting from changes in many contexts accumulated in greater stress for students with pre-existing mental health concerns, which in turn enhanced their psychological risk. For example, during the transition to online learning, these students felt isolated in their academic environment and frequently mentioned the challenges of connecting virtually with professors and peers and were dissatisfied with changes in course assessments (e.g., reduction in group-based assignments). These challenges resulted in increasing distress, created perceived roadblocks to academic success, and bolstered existing feelings of isolation. The heightened sense of isolation derived from changes in many contexts, coupled with limited previous experience managing distress, likely enhanced the distress experienced by students without pre-existing mental health concerns.

Although students with pre-existing mental health concerns also experienced pandemic related stress, they commonly spoke about how changes due to the COVID-19 pandemic offered some positive benefits. Overall, students with pre-existing mental health concerns indicated that they were less adversely influenced by COVID-19 specific changes relative to students without pre-existing mental health concerns. For example, students with pre-existing mental health concerns found that while some aspects of online learning were more difficult, many components enhanced their overall academic experience. These students felt more comfortable participating in class, were able to focus better in online learning environments, and preferred the reduction in group-based assignments more so than students without pre-existing mental health concerns. COVID-19 public health measures, specifically stay-at-home orders, also led to an increase in time alone, which many students with pre-existing mental health concerns indicated they used to focus on self-development or practice self-care activities. These findings are consistent with a broader literature suggesting that there may have been benefits associated with the pandemic for some individuals (Cornell et al., 2021; Schmiedeberg & Thönnissen, 2021; Stallard et al., 2021; Williams et al., 2021).

Given that the identification of positive outcomes was largely unique to students with pre-existing mental health concerns, it is possible that their ability to focus on positives derived from the pandemic contributed to their mental health maintenance (as also outlined by Vuletić et al., 2021). However, it is also possible that students with pre-existing mental health concerns fared well because they had general preferences for changing academic and social contexts, which in turn made coping more manageable. For example, across the interviews some students with pre-existing mental health concerns suggested that they preferred to spend time alone prior to the COVID-19 pandemic, and so it is possible that the social isolation experienced by many during the pandemic was not as impactful for them (Hamza et al., 2020). Further, many students with pre-existing mental health concerns preferred online learning contexts, as previously outlined. It is therefore also possible that these students maintained consistent levels of mental health because many of the pandemic-related challenges were not as consequential for them, and at times were actually favourable.

### Limitations and Future Directions

The present study has many notable strengths, including the focus on the impact of the COVID-19 pandemic among emerging adults enrolled in post-secondary school, and taking an in-depth qualitative analytic approach to understand students’ unique perspectives of their experiences during the pandemic. However, there are also several limitations to highlight. First, the sample was limited to students attending one large urban university who were currently living in the surrounding area. Students who were required to relocate significantly (i.e., to a different country or province/state) were not included in this study and may have different experiences than those currently living in Toronto, Ontario. Greater understanding of students who participated in the post-secondary context remotely is an important avenue for future research. Practitioners registered in the province of Ontario (such as those situated at the University of Toronto) would be unable to provide care out of province given licensing regulations, which may limit mental health access for these students. The sample was also primarily female, East Asian, South Asian, or White, and of middle to upper-class economic backgrounds. The views of students expressed in this present study are contextually relevant, and tied to the gender, ethnic and economic identities of the participants interviewed. Understanding the impact of the pandemic on more diverse samples, in which intersectionality between mental health and other marginalized identities can be further explored, represents an important extension for future research. In addition, emerging evidence suggests that individuals diagnosed with certain psychiatric disorders (e.g., eating disorders, post-traumatic stress disorder) may be more vulnerable to the impacts of the COVID-19 pandemic than others. Thus, it is therefore possible that results may differ for students with mental health concerns not assessed in the present study (Manchia et al., 2021; Taquet et al., 2021).

It is also important to note that all interviews were conducted in early 2021, and student experiences may have changed over time as the pandemic evolved. It is possible that some students developed effective coping strategies across the COVID-19 pandemic, and that the present results may be primarily reflective of early- to mid-pandemic experiences. Moreover, we did not measure whether participants had sought professional support before or during the pandemic. Professional support may have been more accessible for students who were already experiencing mental health concerns prior to the pandemic and would likely influence an individual’s perceived ability to manage pandemic-related distress. Gender differences in service utilization should also be acknowledged when interpreting the present results, given that males are often less likely to seek out mental health services than females (Cadigan et al., 2019). We also did not utilize a clinical, standardized assessment to evaluate participant mental health; instead, we focused on individual perceptions of changes in their mental health and well-being over the COVID-19 pandemic. Capturing students’ subjective experiences is critical to understanding how individuals uniquely respond to novel stressors, such as the pandemic, and also to understand nuances in lived experiences, but does little to quantify changes in mental health and well-being.

Future research may benefit from looking at longitudinal changes in stress and coping abilities across the COVID-19 pandemic, taking into account help-seeking experiences, as well as greater consideration of individual differences among emerging adults enrolled in university. For example, future research should thoroughly investigate the influence that social and/or academic isolation can have on post-secondary student well-being over time, with a specific focus on the potential differential influence for students with and without pre-existing mental health concerns.

## Conclusions and Implications

In the present study, we sought to explore and understand the differential impacts of the COVID-19 pandemic on students with and without pre-existing mental health concerns (Hamza et al., 2020; Meda et al., 2021). It was found that students without pre-existing mental health concerns had a more difficult time adjusting to the pandemic than students who already had mental health challenges. The present study also elucidated several factors that may contribute to the discrepancy in mental health impacts between students with and without pre-existing mental health concerns. Specifically, students with pre-existing mental health concerns reported leveraging existing coping strategies and identified positive experiences from the pandemic. In contrast, students without pre-existing mental health concerns reported more difficulty adapting and accessing coping strategies in the context of the pandemic, and experienced heightened social and academic isolation. These students also found online learning more challenging as compared to students with pre-existing mental health concerns, who seemed to prefer more independent learning. The present findings suggest that the students most at-risk for mental health deterioration were those without pre-existing mental health concerns, who experienced the greatest increase in novel stressors and had difficulty adapting coping strategies when their typical means of managing stress were restricted.

Given the initial concerns that students with pre-existing mental health concerns would be most vulnerable in the context of the COVID-19 pandemic, the present findings accentuate a need to be cautious when considering who is most at-risk for increasing distress in times of novel stress. It is important to acknowledge that previous experiences managing mental health concerns may provide individuals with the opportunity to learn how to manage stress and build resilience, which in turn may support adjustment during challenging times as opposed to further exacerbating symptoms of distress. The results of the present study suggest that it is imperative to equip all students, regardless of their existing mental health status, with the opportunity to develop a repertoire of coping strategies. This will ensure that students will have access to strategies in the presence of new or unanticipated stressors, as well as to manage the ongoing effects of the pandemic. Further, results underscore that universities will not only need to continue to support students with pre-existing mental health concerns, but should also expand supports for students who are beginning to struggle in the context of the pandemic.

Additionally, it is important for post-secondary institutions to continue to acknowledge the diverse learning needs of students and tailor course delivery when possible. Findings illustrate that some students thrive in face-to-face learning environments whereas other students may prefer online learning options; in response, institutions may need to look for novel ways to support the learning needs of all students beyond the COVID-19 pandemic. For example, institutions could explore the implementation of hybrid courses that include both in-person and online learning options to cater to the preferences of all students and offer more flexibility in learning environments. While still novel, emerging evidence on hybrid approaches indicate pedagogical and organizational benefits for students, staff, and institutions (Raes et al., 2020).

Finally, some students reported that the lack of academic community during the pandemic negatively impacted their classroom experiences and sense of well-being. This finding underscores that it is important for colleges and universities to consider implementing different programming (i.e., safe socialization opportunities), in order to holistically support students. All students in the present study emphasized how much they appreciated academic institutions providing supportive and accommodating learning environments during a new and challenging time, underscoring the integral, and unique, role post-secondary institutions play in the lives of emerging adults.

## References

[bibr1-21676968221121590] American College Health Association. (2019). American college health association-national college health assessment II: Canadian consortium executive summary spring 2019. American College Health Association.

[bibr2-21676968221121590] Arnett J. J. Žukauskienė R. Sugimura K. (2014). The new life stage of emerging adulthood at ages 18–29 years: Implications for mental health. The Lancet Psychiatry, 1(7), 569–576. 10.1016/S2215-0366(14 26361316

[bibr3-21676968221121590] Assarroudi A. Nabavi F. H. Armat M. R. Ebadi A. Vaismoradi M. (2018). Directed qualitative content analysis: The description and elaboration of its underpinning methods and data analysis process. Journal of Research in Nursing, 23(1), 42–55. 10.1177/1744987117741667 34394406 PMC7932246

[bibr4-21676968221121590] Auerbach R. P. Alonso J. Axinn W. G. Cuijpers P. Ebert D. D. Green J. G. Hwang I. Kessler R. C. Liu H. Mortier P. Nock M. K. Pinder-Amaker S. Sampson N. A. Aguilar-Gaxiola S. Al-Hamzawi A. Andrade L. H. Benjet C. Caldas-de-Almeida J. M. Demyttenaere K. Bruffaerts R. (2016). Mental disorders among college students in the world health organization world mental health surveys. Psychological Medicine, 46(14), 2955–2970. 10.1017/S0033291716001665 27484622 PMC5129654

[bibr5-21676968221121590] Browning M. H. E. M. Larson L. R. Sharaievska I. Rigolon A. McAnirlin O. Mullenbach L. Cloutier S. Vu T. M. Thomsen J. Reigner N. Metcalf E. C. D’Antonio A. Helbich M. Bratman G. N. Alvarez H. O. (2021). Psychological impacts from COVID-19 among university students: Risk factors across seven states in the United States. Plos One, 16(1), Article e0245327. 10.1371/journal.pone.0245327 33411812 PMC7790395

[bibr6-21676968221121590] Bureau of Labor Statistics, U.S. Department of Labor. (2020). 66.2 percent of 2019 high school graduates enrolled in college in October 2019 (The Economics Daily). https://www.bls.gov/opub/ted/2020/66-point-2-percent-of-2019-high-school-graduates-enrolled-in-college-in-october-2019.htm

[bibr7-21676968221121590] Cadigan J. M. Lee C. M. Larimer M. E. (2019). Young adult mental health: A prospective examination of service utilization, perceived unmet service needs, attitudes, and barriers to service use. Prevention Science, 20(3), 366–376. 10.1007/s11121-018-0875-8 29411197 PMC6081266

[bibr8-21676968221121590] Cao W. Fang Z. Hou G. Han M. Xu X. Dong J. Zheng J. (2020). The psychological impact of the COVID-19 epidemic on college students in China. Psychiatry Research, *287*, 112934. 10.1016/j.psychres.2020.112934. 32229390 PMC7102633

[bibr9-21676968221121590] Clarke V. Braun V. (2017). Thematic analysis. The Journal of Positive Psychology, 12(3), 297–298. 10.1080/17439760.2016.1262613

[bibr10-21676968221121590] Cleary M. Horsfall J. Hayter M. (2014). Data collection and sampling in qualitative research: Does size matter? Journal of Advanced Nursing, 70(3), 3–475. 10.1111/jan.12163 24450874

[bibr11-21676968221121590] Cornell S. Nickel B. Cvejic E. Bonner C. McCaffery K. J. Ayre J. Copp T. Batcup C. Isautier J. Dakin T. Dodd R. Judd J. (2021). Positive outcomes associated with the COVID-19 pandemic in Australia. Health Promotion Journal of Australia. 33(2), 311–319. 10.1002/hpja.494 33864299 PMC8250613

[bibr12-21676968221121590] Czysz A. H. Nandy K. Hughes J. L. Minhajuddin A. Chin Fatt C. R. Trivedi M. H. (2021). Impact of the COVID-19 pandemic on adults with current and prior depression: Initial findings from the longitudinal Texas RAD study. Journal of Affective Disorders, 294, 103–108. 10.1016/j.jad.2021.06.071 34274785 PMC8433599

[bibr13-21676968221121590] Dazzi T. Gribble R. Wessely S. Fear N. T. (2014). Does asking about suicide and related behaviours induce suicidal ideation? What is the evidence? Psychological Medicine, 44(16), 3361–3363. 10.1017/S0033291714001299 24998511

[bibr14-21676968221121590] Druss B. G. (2020). Addressing the COVID-19 pandemic in populations with serious mental illness. JAMA Psychiatry, 77(9), 891. 10.1001/jamapsychiatry.2020.0894 32242888

[bibr15-21676968221121590] Eaton W. W. Smith C. Ybarra M. Muntaner C. Tien A. (2004). Center for epidemiologic studies depression scale: Review and revision (CESD and CESD-R). In The use of psychological testing for treatment planning and outcomes assessment: Instruments for adults. (Vol. 3, pp. 363–377). Lawrence Erlbaum Associates.

[bibr16-21676968221121590] Elmer T. Mepham K. Stadtfeld C. (2020). Students under lockdown: Comparisons of students’ social networks and mental health before and during the COVID-19 crisis in Switzerland. Plos One, 15(7), Article e0236337. 10.1371/journal.pone.0236337 32702065 PMC7377438

[bibr17-21676968221121590] Elo S. Kyngas H. (2008). The qualitative content analysis process. Journal of Advanced Nursing, 62(1), 107–115. 10.1111/j.1365-2648.2007.04569.x 18352969

[bibr18-21676968221121590] Fancourt D. Steptoe A. Bu F. (2021). Trajectories of anxiety and depressive symptoms during enforced isolation due to COVID-19 in england: A longitudinal observational study. The Lancet Psychiatry, 8(2), 141–149. 10.1016/S2215-0366(20 33308420 PMC7820109

[bibr19-21676968221121590] Farris S. G. Kibbey M. M. Fedorenko E. J. DiBello A. M. (2021). A qualitative study of COVID-19 distress in university students. Emerging Adulthood, 9(5), 462–478, 10.1177/21676968211025128

[bibr20-21676968221121590] Fiorillo A. Gorwood P. (2020). The consequences of the COVID-19 pandemic on mental health and implications for clinical practice. European Psychiatry, 63(1), Article e32. 10.1192/j.eurpsy.2020.35 32234102 PMC7156565

[bibr21-21676968221121590] Francis J. J. Johnston M. Robertson C. Glidewell L. Entwistle V. Eccles M. P. Grimshaw J. M. (2010). What is an adequate sample size? Operationalising data saturation for theory-based interview studies. Psychology & Health, 25(10), 1229–1245. 10.1080/08870440903194015 20204937

[bibr22-21676968221121590] Galea S. Merchant R. M. Lurie N. (2020). The mental health consequences of COVID-19 and physical distancing: The need for prevention and early intervention. JAMA Internal Medicine, 180(6), 817. 10.1001/jamainternmed.2020.1562 32275292

[bibr23-21676968221121590] Gardner K. Qualter P. (2009). Reliability and validity of three screening measures of borderline personality disorder in a nonclinical population. Personality and Individual Differences, 46(5–6), 636–641. 10.1016/j.paid.2009.01.005

[bibr24-21676968221121590] Golestaneh L. Neugarten J. Fisher M. Billett H. H. Gil M. R. Johns T. Yunes M. Mokrzycki M. H. Coco M. Norris K. C. Perez H. R. Scott S. Kim R. S. Bellin E. (2020). The association of race and COVID-19 mortality. EClinicalMedicine, *25*, 100455. 10.1016/j.eclinm.2020.100455 32838233 PMC7361093

[bibr25-21676968221121590] Government of Ontario. (2021, February 8). Ontario extending stay-at-home order across most of the province to save lives [Press Release]. https://news.ontario.ca/en/release/60261/ontario-extending-stay-at-home-order-across-most-of-the-province-to-save-lives

[bibr26-21676968221121590] Gunnell D. Appleby L. Arensman E. Hawton K. John A. Kapur N. Khan M. O’Connor R. C. Pirkis J Caine E. D. Chan L. F. Chang S. S. Chen Y. Y. Christensen H. Dandona R. Eddleston M. Erlangsen A. Yip P. S. (2020). Suicide risk and prevention during the COVID-19 pandemic. Lancet Psychiatry, 2019(20), 1–3. 10.1016/S2215-0366(20 PMC7173821 32330430

[bibr27-21676968221121590] Hagman B. T. (2016). Performance of the AUDIT in detecting DSM-5 alcohol use disorders in college students. Substance Use & Misuse, 51(11), 1521–1528. 10.1080/10826084.2016.1188949 27438676

[bibr28-21676968221121590] Halliburton A. E. Hill M. B. Dawson B. L. Hightower J. M. Rueden H. (2021). Increased stress, declining mental health: Emerging adults’ experiences in college during COVID-19. Emerging Adulthood, 9(5), 433–448. 10.1177/21676968211025348

[bibr29-21676968221121590] Hamm M. E. Brown P. J. Karp J. F. Lenard E. Cameron F. Dawdani A. Lavretsky H. Miller J. P. Mulsant B. H. Pham V. T. Reynolds C. F. Roose S. P. Lenze E. J. (2020). Experiences of American older adults with pre-existing depression during the beginnings of the COVID-19 pandemic: A multicity, mixed-methods study. The American Journal of Geriatric Psychiatry, 28(9), 924–932. 10.1016/j.jagp.2020.06.013 32682619 PMC7305766

[bibr30-21676968221121590] Hamza C. A. Ewing L. Heath N. L. Goldstein A. L. (2020). When social isolation is nothing new: A longitudinal study on psychological distress during COVID-19 among university students with and without preexisting mental health concerns. Canadian Psychology/Psychologie Canadienne, 62(1), 20–30. 10.1037/cap0000255

[bibr31-21676968221121590] Hotez E. Gragnani C. M. Fernandes P. Rosenau K. A. Chopra A. Chung A. Grassian J. Huynh S. Jackson T. Jimenez K. Jue E. Le N. Lenghong J. Lopez A. Lopez L. Omo-Sowho P. Pennington K. Tirado R. Kuo A. (2021). Capturing the experiences and challenges of emerging adults in college during the COVID-19 pandemic. Cureus. 13(8), Article e17605, 10.7759/cureus.17605 34646656 PMC8483390

[bibr32-21676968221121590] Hsieh H.-F. Shannon S. E. (2005). Three approaches to qualitative content analysis. Qualitative Health Research, 15(9), 1277–1288. 10.1177/1049732305276687 16204405

[bibr33-21676968221121590] Iob E. Steptoe A. Fancourt D. (2020). Abuse, self-harm and suicidal ideation in the UK during the COVID-19 pandemic. The British Journal of Psychiatry, 217(4), 543–546. 10.1192/bjp.2020.130 32654678 PMC7360935

[bibr34-21676968221121590] Kibbey M. M. Fedorenko E. J. Farris S. G. (2021). Anxiety, depression, and health anxiety in undergraduate students living in initial US outbreak “hotspot” during COVID-19 pandemic. Cognitive Behaviour Therapy, 50(5), 409–421. 10.1080/16506073.2020.1853805 33433271

[bibr35-21676968221121590] Kujawa A. Green H. Compas B. E. Dickey L. Pegg S. (2020). Exposure to COVID-19 pandemic stress: Associations with depression and anxiety in emerging adults in the United States. Depression and Anxiety, 37(12), 1280–1288. 10.1002/da.23109 33169481 PMC13347965

[bibr36-21676968221121590] Kyngäs H. Kääriäinen M. Elo S. (2019). Trustworthiness in the context of qualitative research. In Kyngäs H. Mikkonen K. Kääriäinen M. (Eds.), The application of content analysis in nursing science. Springer Nature.

[bibr37-21676968221121590] Lederer A. M. Hoban M. T. Lipson S. K. Zhou S. Eisenberg D. (2021). More than inconvenienced: The unique needs of U.S. college students during the COVID-19 pandemic. Health Education & Behavior, 48(1), 14–19. 10.1177/1090198120969372 33131325 PMC8356799

[bibr38-21676968221121590] Li X. Fu P. Fan C. Zhu M. Li M. (2021). COVID-19 stress and mental health of students in locked-down colleges. International Journal of Environmental Research and Public Health, 18(2), 771. 10.3390/ijerph18020771 PMC7831318 33477595

[bibr39-21676968221121590] Lincoln Y. S. Guba E. G. (1985). Naturalistic inquiry. SAGE Publications.

[bibr40-21676968221121590] López-Castro T. Brandt L. Anthonipillai N. J. Espinosa A. Melara R. (2021). Experiences, impacts and mental health functioning during a COVID-19 outbreak and lockdown: Data from a diverse New York City sample of college students. Plos One, 16(4), Article e0249768. 10.1371/journal.pone.0249768 33826654 PMC8026074

[bibr41-21676968221121590] Manchia M. Gathier A. W. Yapici-Eser H. Schmidt M. V. de Quervain D. van Amelsvoort T. Bisson J. I. Cryan J. F. Howes O. D. Pinto L. van der Wee N. J. Domschke K. Branchi I. Vinkers C. H. (2021). The impact of the prolonged COVID-19 pandemic on stress resilience and mental health: A critical review across waves. European Neuropsychopharmacology, 55, 22–83. 10.1016/j.euroneuro.2021.10.864 34818601 PMC8554139

[bibr42-21676968221121590] Mathias K. Rawat M. Philip S. Grills N. (2020). “We’ve got through hard times before”: Acute mental distress and coping among disadvantaged groups during COVID-19 lockdown in North India - a qualitative study. International Journal for Equity in Health, 19(1), 224. 10.1186/s12939-020-01345-7 33334344 PMC7745174

[bibr43-21676968221121590] Meda N. Pardini S. Slongo I. Bodini L. Zordan M. A. Rigobello P. Visioli F. Novara C. (2021). Students’ mental health problems before, during, and after COVID-19 lockdown in Italy. Journal of Psychiatric Research, 134, 69–77. 10.1016/j.jpsychires.2020.12.045 33360865

[bibr44-21676968221121590] Mikkonen K. Kyngäs H. (2019). Content analysis in mixed methods research. In Kyngäs H. Mikkonen K. Kääriäinen M. (Eds.), The application of content analysis in nursing science research. Springer Nature.

[bibr45-21676968221121590] Murphy L. Markey K. O’ Donnell C. Moloney M. Doody O. (2021). The impact of the COVID-19 pandemic and its related restrictions on people with pre-existent mental health conditions: A scoping review. Archives of Psychiatric Nursing, 35(4), 375–394. 10.1016/j.apnu.2021.05.002 34176579 PMC9759111

[bibr46-21676968221121590] Odriozola-González P. Planchuelo-Gómez Á. Irurtia M. J. de Luis-García R. (2020). Psychological effects of the COVID-19 outbreak and lockdown among students and workers of a Spanish university. Psychiatry Research, *290*, 113108. 10.1016/j.psychres.2020.113108 32450409 PMC7236679

[bibr47-21676968221121590] Oswalt S. B. Lederer A. M. Chestnut-Steich K. Day C. Halbritter A. Ortiz D. (2020). Trends in college students’ mental health diagnoses and utilization of services, 2009–2015. Journal of American College Health, 68(1), 41–51. 10.1080/07448481.2018.1515748 30355071

[bibr48-21676968221121590] Pan K.-Y. Kok A. A. L. Eikelenboom M. Horsfall M. Jörg F. Luteijn R. A. Rhebergen D. van Oppen P. Giltay E. J. Penninx B. W. J. H. (2021). The mental health impact of the COVID-19 pandemic on people with and without depressive, anxiety, or obsessive-compulsive disorders: A longitudinal study of three Dutch case-control cohorts. The Lancet Psychiatry, 8(2), 121–129. 10.1016/S2215-0366(20 33306975 PMC7831806

[bibr49-21676968221121590] Patel J. A. Nielsen F. B. H. Badiani A. A. Assi S. Unadkat V. A. Patel B. Ravindrane R. Wardle H. (2020). Poverty, inequality and COVID-19: The forgotten vulnerable. Public Health, *183*, 110–111. 10.1016/j.puhe.2020.05.006 PMC7221360 32502699

[bibr50-21676968221121590] Patterson Z. R. Gabrys R. L. Prowse R. K. Abizaid A. B. Hellemans K. G. C. McQuaid R. J. (2021). The influence of COVID-19 on stress, substance use, and mental health among postsecondary students. Emerging Adulthood, 9(5), 516–530. 10.1177/21676968211014080

[bibr51-21676968221121590] Pierce M. Hope H. Ford T. Hatch S. Hotopf M. John A. Kontopantelis E. Webb R. Wessely S. McManus S. Abel K. M. (2020). Mental health before and during the COVID-19 pandemic: A longitudinal probability sample survey of the UK population. The Lancet Psychiatry, 7(10), 883–892. 10.1016/S2215-0366(20 32707037 PMC7373389

[bibr52-21676968221121590] Pinkham A. E. Ackerman R. A. Depp C. A. Harvey P. D. Moore R. C. (2020). A longitudinal investigation of the effects of the COVID-19 pandemic on the mental health of individuals with pre-existing severe mental illnesses. Psychiatry Research, *294*, 113493. 10.1016/j.psychres.2020.113493 33038789 PMC7528831

[bibr53-21676968221121590] Prati G. Mancini A. D. (2021). The psychological impact of COVID-19 pandemic lockdowns: A review and meta-analysis of longitudinal studies and natural experiments. Psychological Medicine, 51(2), 201–211. 10.1017/S0033291721000015 33436130 PMC7844215

[bibr54-21676968221121590] Raes A. Detienne L. Windey I. Depaepe F. (2020). A systematic literature review on synchronous hybrid learning: Gaps identified. Learning Environments Research, 23(3), 269–290. 10.1007/s10984-019-09303-z

[bibr55-21676968221121590] Ray E. C. Perko A. Oehme K. Arpan L. Clark J. Bradley L. (2021). Freshmen anxiety and COVID-19: Practical implications from an online intervention for supporting students affected by health inequities. Journal of American College Health, 1–10. 10.1080/07448481.2021.1965610 34449301

[bibr56-21676968221121590] Sahu P. (2020). Closure of universities due to coronavirus disease 2019 (COVID-19): Impact on education and mental health of students and academic staff. Cureus. 12(4), Article e7541. 10.7759/cureus.7541 32377489 PMC7198094

[bibr57-21676968221121590] Saunders J. B. Aasland O. G. Babor T. F. De La Fuente J. R. Grant M. (1993). Development of the alcohol use disorders identification test (AUDIT): WHO collaborative project on early detection of persons with harmful alcohol consumption-II. Addiction, 88(6), 791–804. 10.1111/j.1360-0443.1993.tb02093.x 8329970

[bibr58-21676968221121590] Schmiedeberg C. Thönnissen C. (2021). Positive and negative perceptions of the COVID-19 pandemic: Does personality play a role? Social Science & Medicine, 276, 113859. 10.1016/j.socscimed.2021.113859 33799202 PMC9756788

[bibr59-21676968221121590] Smith J. A. Judd J. (2020). COVID-19: Vulnerability and the power of privilege in a pandemic. Health Promotion Journal of Australia, 31(2), 158–160. 10.1002/hpja.333 32197274 PMC7165578

[bibr60-21676968221121590] Son C. Hegde S. Smith A. Wang X. Sasangohar F. (2020). Effects of COVID-19 on college students’ mental health in the United States: Interview survey study. Journal of Medical Internet Research, 22(9), Article e21279. 10.2196/21279 32805704 PMC7473764

[bibr61-21676968221121590] Spitzer R. L. Kroenke K. Williams J. B. Lo B. (2006). A brief measure for assessing generalized anxiety disorder: The GAD-7. Archives of Internal Medicine, 166(10), 1092–1097. 10.1001/archinte.166.10.1092 16717171

[bibr62-21676968221121590] Sriken J. Johnsen S. T. Smith H. Sherman M. F. Erford B. T. (2022). Testing the factorial validity and measurement invariance of college student scores on the Generalized Anxiety Disorder (GAD-7) scale across gender and race. Measurement and Evaluation in Counseling and Development, 55(1), 1–16. 10.1080/07481756.2021.1902239

[bibr63-21676968221121590] Stallard P. Pereira A. I. Barros L. (2021). Post-traumatic growth during the COVID-19 pandemic in carers of children in Portugal and the UK: Cross-sectional online survey. The British Journal of Psychiatry, 7(1), Article e37. 10.1192/bjo.2021.1 PMC7844169 33468270

[bibr64-21676968221121590] Statistics Canada. (2017). Census profile, 2016 census. https://www12.statcan.gc.ca/census-recensement/2016/dp-pd/prof/index.cfm?Lang=E

[bibr65-21676968221121590] Statistics Canada. (2019a). Classification of programs and credentials—8—undergraduate program. https://www23.statcan.gc.ca/imdb/p3VD.pl?Function=getVD&TVD=1252482&CVD=1252483&CPV=8&CST=23072019&CLV=1&MLV=2

[bibr66-21676968221121590] Statistics Canada. (2019b). Table 37-10-0086-01 Postsecondary enrolments, by status of student in Canada, country of citizenship and gender. https://www150.statcan.gc.ca/t1/tbl1/en/tv.action?pid=3710008601&pickMembers/5B0/5D=1.1&pickMembers/5B1/5D=2.1

[bibr67-21676968221121590] Taquet M. Geddes J. R. Luciano S. Harrison P. J. (2021). Incidence and outcomes of eating disorders during the COVID-19 pandemic. The British Journal of Psychiatry, 3(5), 262–264. 10.1192/bjp.2021.105 PMC7612698 35048812

[bibr68-21676968221121590] Van Dam N. T. Earleywine M. (2011). Validation of the center for epidemiologic studies depression scale—revised (CESD-R): Pragmatic depression assessment in the general population. Psychiatry Research, 186(1), 128–132. 10.1016/j.psychres.2010.08.018 20843557

[bibr69-21676968221121590] van den Berg Y. H. M. Burk W. J. Cillessen A. H. N. Roelofs K. (2021). Emerging adults’ mental health during the COVID-19 pandemic: A prospective longitudinal study on the importance of social support. Emerging Adulthood, 9(5), 618–630, 10.1177/21676968211039979 34925969 PMC8669206

[bibr70-21676968221121590] van Zyl L. E. Rothmann S. Zondervan-Zwijnenburg M. A. J. (2021). Longitudinal trajectories of study characteristics and mental health before and during the COVID-19 lockdown. Frontiers in Psychology, *12*, 633533. 10.3389/fpsyg.2021.633533 33776857 PMC7987834

[bibr71-21676968221121590] Vuletić T. Ignjatović N. Stanković B. Ivanov A. (2021). “Normalizing” everyday life in the state of emergency: Experiences, well-being and coping strategies of emerging adults in Serbia during the first wave of the COVID-19 pandemic. Emerging Adulthood, 9(5), 583–601, 10.1177/21676968211029513

[bibr72-21676968221121590] Watkins-Martin K. Orri M. Pennestri M.-H. Castellanos-Ryan N. Larose S. Gouin J.-P. Ouellet-Morin I. Chadi N. Philippe F. Boivin M. Tremblay R. E. Côté S. Geoffroy M.-C. (2021). Depression and anxiety symptoms in young adults before and during the COVID-19 pandemic: Evidence from a Canadian population-based cohort. Annals of General Psychiatry, 20(1), 42. 10.1186/s12991-021-00362-2 34496901 PMC8424412

[bibr73-21676968221121590] Williams L. Rollins L. Young D. Fleming L. Grealy M. Janssen X. Kirk A. MacDonald B. Flowers P. (2021). What have we learned about positive changes experienced during COVID-19 lockdown? Evidence of the social patterning of change. Plos One, 16(1), Article e0244873. 10.1371/journal.pone.0244873 33400700 PMC7785245

[bibr74-21676968221121590] Xiong J. Lipsitz O. Nasri F. Lui L. M. W. Gill H. Phan L. Chen-Li D. Iacobucci M. Ho R. Majeed A. McIntyre R. S. (2020). Impact of COVID-19 pandemic on mental health in the general population: A systematic review. Journal of Affective Disorders, *277*, 55–64. 10.1016/j.jad.2020.08.001 PMC7413844 32799105

[bibr75-21676968221121590] Yao H. Chen J.-H. Xu Y.-F. (2020). Patients with mental health disorders in the COVID-19 epidemic. The Lancet Psychiatry, 7(4), Article e21. 10.1016/S2215-0366(20 32199510 PMC7269717

[bibr76-21676968221121590] Zanarini M. C. Vujanovic A. A. Parachini E. A. Boulanger J. L. Frankenburg F. R. Hennen J. (2003). A screening measure for BPD: The McLean screening instrument for borderline personality disorder (MSI-BPD). Journal of Personality Disorders, 17(6), 568–573. 10.1521/pedi.17.6.568.25355 14744082

[bibr77-21676968221121590] Zimmer-Gembeck M. J. Skinner E. A. (2016). The development of coping: Implications for psychopathology and resilience. In Developmental psychopathology (pp. 1–61). John Wiley & Sons Inc. 10.1002/9781119125556.devpsy410

